# Nasojugal Flap with Dermal Pennant for Reconstructive of Lower Lid Defect

**DOI:** 10.29252/wjps.8.2.245

**Published:** 2019-05

**Authors:** Mojtaba Nasiri, Mohammad H Kardar

**Affiliations:** 1 School of Medicine, Shahroud University of Medical Sciences, Shahroud, Iran;; 2 Department of Plastic Surgery, School of Medicine, Shahroud University of Medical Sciences, Shahroud, Iran

**Keywords:** Nasojugal flap, Dermal pennant, Lower lid

## Abstract

**BACKGROUND:**

Basal cell carcinomas (BCCs) are locally invasive periocular skin cancers affecting lower eyelids more than upper eyelids. The purpose of this study was to describe techniques used for lower eyelid reconstruction after extended excision of BCC.

**METHODS:**

Eight referred patients with BCC who underwent lower eyelid reconstruction were enrolled. The tumor was surgically excised with sufficient margins by one surgeon. Defects were repaired by subdermal tunnel between lateral border of defect and insertion site of lateral cantus.

**RESULTS:**

Eight patients aged 45 to 75 years were followed up for 6 months. After follow up, adequate viability of the grafts, satisfactory functional and good cosmetic results was noticed in all patients. One patient complained of irritation at the site of surgery. No total or partial necrosis, hematoma, or infection were observed in flaps, and no additional surgery was needed.

**CONCLUSION:**

The present novel surgical procedure was useful to close full thickness defects in the lower lid to preserve the function of the lower eyelid and a good aesthetic outcome.

## INTRODUCTION

With increasing the age of the population, skin cancer incidence is increasing. Significant proportion of skin cancers occur on the face. Basal cell carcinoma (BCC) accounts for approximately 90% of eyelid tumors and most frequently involve the lower eyelid.^1^^-^^3^ Two common causes of eyelid defects that required surgical reconstruction are eyelid tumor excision and trauma. Several techniques can be used to reconstruct lower lid defects. For defects of less than 25% of upper and lower eyelid, lateral canthotomy, for defects of more than 25% of lower eyelid up to entire, mustard method (or Tenzel variation) and for total or near total defects of lower eyelid the nasojugal flap of Tessier with nasal chondromucosal graft are execute methods.^4^

Ectropion is most commonly observed as involutional change associated with horizontal laxity of the involved eyelid. Ectropion can be assortment into 5 types of congenital, mechanical, senile, paralytic andcicatricial.^5^ Ectropion risk is dependent on factors of defect size and depth; type of reconstructive procedure performed; preexisting laxity; and position of the maxilla in relation to the orbit.^6^^-^^8^ We designed a new method and use of nasojugal flap with pennant for lower eyelid reconstruction for prevention and reduction of the ectropion rate. 

## MATERIALS AND METHODS

This is prospective single center study. All patients underwent eyelid surgery at our hospital in Tehran, Iran. All of the cases were affected by BCC. The reconstruction involved the lower lid in all cases. All patients underwent radical resection of the neoplastic lesion, as confirmed by extemporaneous histological examination of several sections to ascertain whether the lateral and deep resection margins were clear. The extent of tissue removed ranged from 1.2 to 4.5 of the length of the eyelid with a full-thickness excision. All patients were seen at 1 week, 4 week and at 3, 6 months after surgery. Collected data included patients’ demographics, defect size and complications.

After standard preparation limits of resection with sufficient margins was marked, nasojugal flap was designed and then the whole field was infiltrated with 1% lidocaine plus epinephrine (1 in 100000). The lesion was resected with optimal margin (which was documented by frozen section) and after proper hemostasis, a subdermal tunnel was made between lateral border of defect and insertion site of lateral cantus. The flap was elevated and its distal part was de epithelialized and then passed through the before mentioned tunnel fixed to periosteum of lateral rim of orbit 1-2 mm. 

Above the insertion site of lateral cantus by 4-0 prolene using a pledge, other parts of the flap was set in and fixed with 5-0 nylon. Donor site repaired primarily. A modification of nasojugal or nasolabial flap was designed by removing only epidermal in 2-3 cm of distal part of the flap, and a dermal pennant was created. After removing the tumor with safe margin, the resulting defect was covered by the flap. A dermal pennant was fixed in the subcutaneous tunnel that was previously created. 

## RESULTS

**Fig. 1:**
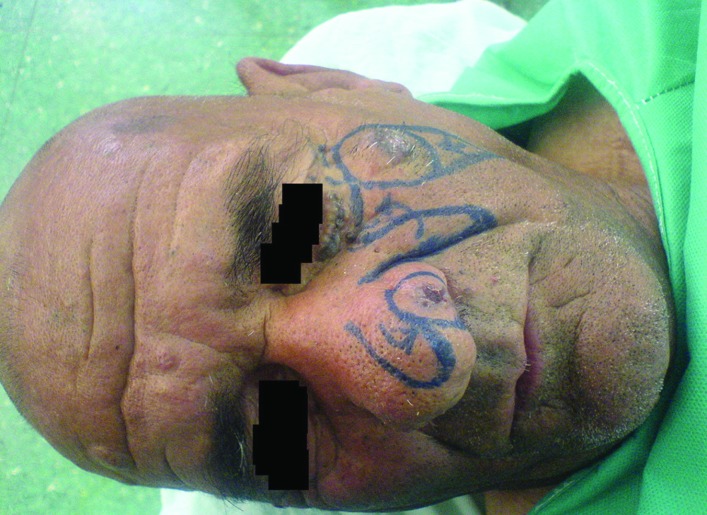
A 72 years old patient with BCC in lower lid

**Fig. 2:**
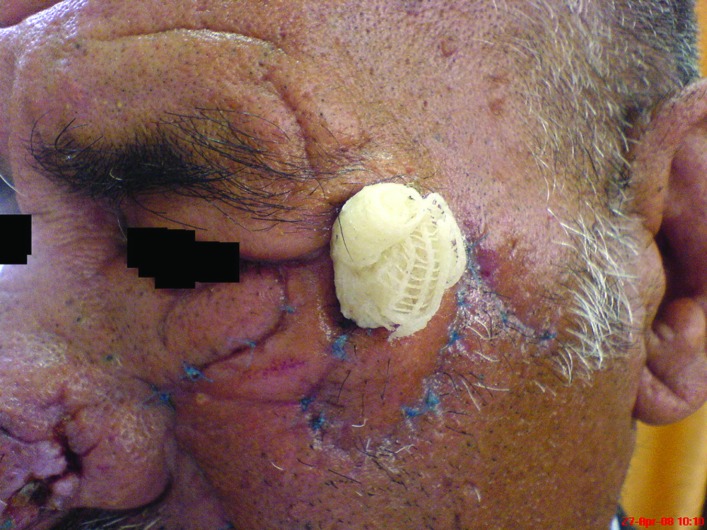
Dermal pennant fixed in place

**Fig. 3:**
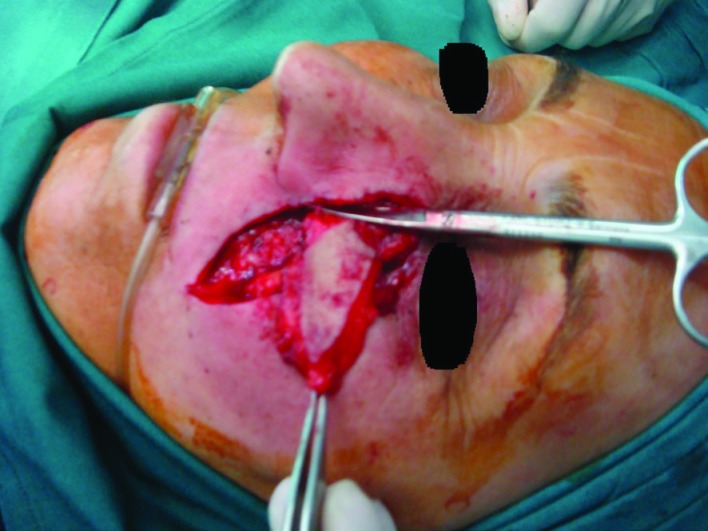
Post-op 2 months later

**Fig. 4:**
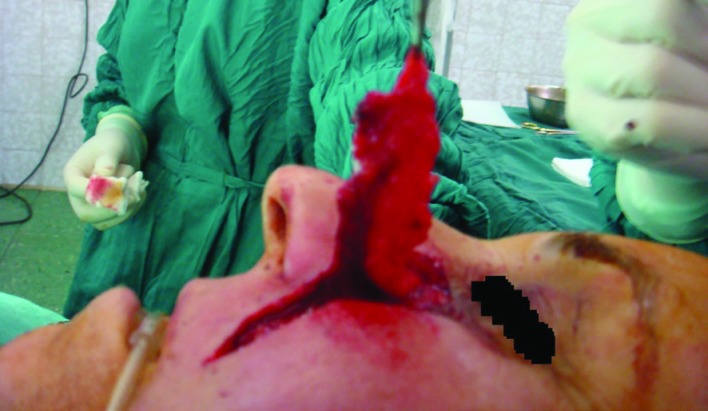
Flap design and elevation

**Fig. 5:**
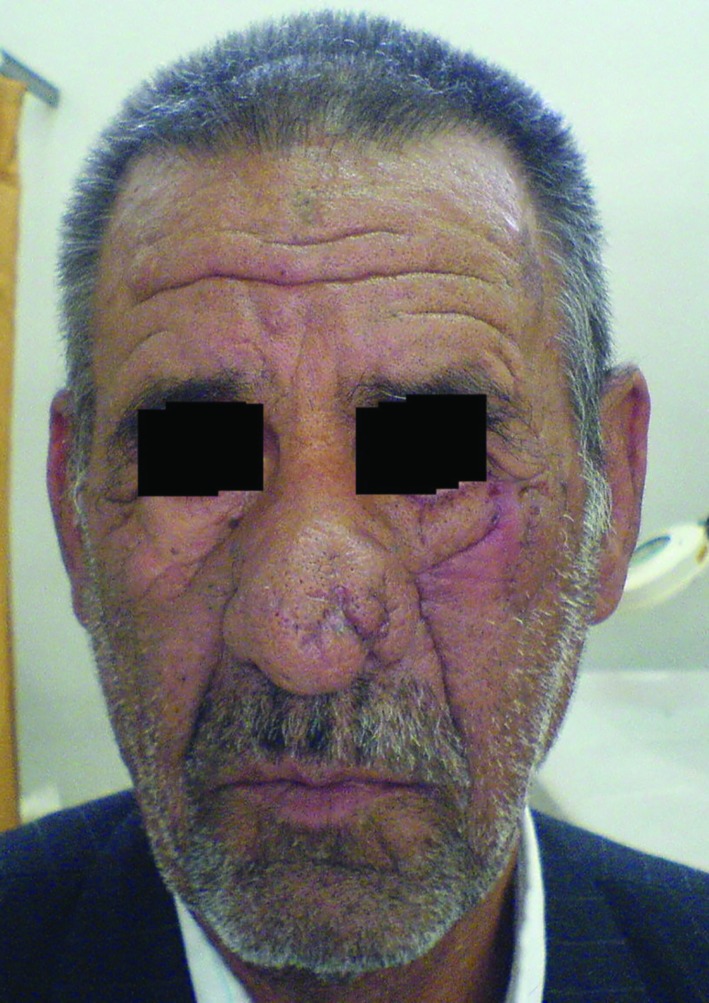
Dermal pennant in the distal end of flap

The eight patients were 6 males and 2 females, aged between 40- 72 years. Two week post-operation, the pledge was removed. Details of patients including age, sex, defect size, complications and follow up periods were mentioned in Table 1. As displayed in the table, at 6 months follow up of 8 patients, there was not any ectropion. Inflammation was observed only in 1 case (Figure 1-5). 

**Table 1:** Details of sex, age, defect size, complication and length of follow up in all patients

**Patient**	**Age**	**Sex**	**Defect size (mm)**	**Complication**	**Follow up time (Month)**
1	65	Female	35*15	None	5
2	60	Female	25*17	None	5
3	45	Male	23*14	None	6
4	72	Male	27*12	Inflammation	6
5	62	Male	17*12	None	6
6	53	Male	30*15	None	6
7	57	Male	27*14	None	6
8	60	Male	20*12	None	6

## DISCUSSION

The new nasojugal flap with dermal pennant used in our study was very helpful and promising in preventing ectropion as one of the most common causes of patient’s dissatisfaction and discomfort. Technics and flap used in our study could protect the structure and function of the lower eyelid. The palatal mucosal graft has previously been used in reconstruction of superficial lower eyelid defect.^9^ In this study similar to our study, no evidence of ectropion was found and tarsus and conjunctiva were fully preserved. Various techniques were applied in reconstruction of upper and lower lid defects including cheek advancement and nasal septal chondromucosal graft. The graft failure and lag ophthalmos were the frequent pitfalls and Epiphora and ptosis were also the major problems. The ectropion was reported in one patient.^10^ Failure to repair nasolacrimal duct was demonstrated as a major drawback after reconstruction of lower lid defects by various types of flaps.^11^

Our modified nasojugal flap with dermal pennant was shown to be a very versatile method for reconstruction of lower lid defects. A dermal pennant has two distinct advantages. First, it prevents ectropion as a major complication. Second, it provides a better contour transition from lower to zygoma. The nasojugal flap itself is lighter than similar flaps like cheek advancement and has a reliable blood supply.^12^ We did not notice any necrosis or ischemic problems in our patients. The scar of nasojugal flap was also very well hidden in nasolabial fold. Drawbacks of flaps including transient edema of pennant in the subcutaneous tunnel and the need for precision and expertise for safe performance and avoiding ischemic problems were noted. Limitation of our study was the number of patients, so use and follow up of the modified flap are recommend on more patients. 

## CONFLICT OF INTEREST

The authors declare no conflict of interest.
